# Concussion As a Multi-Scale Complex System: An Interdisciplinary Synthesis of Current Knowledge

**DOI:** 10.3389/fneur.2017.00513

**Published:** 2017-09-28

**Authors:** Erin S. Kenzie, Elle L. Parks, Erin D. Bigler, Miranda M. Lim, James C. Chesnutt, Wayne Wakeland

**Affiliations:** ^1^Systems Science Program, Portland State University, Portland, OR, United States; ^2^Department of Psychology and Neuroscience Center, Brigham Young University, Provo, UT, United States; ^3^Sleep Disorders Clinic, Division of Hospital and Specialty Medicine, Veterans Affairs Portland Health Care System, Portland, OR, United States; ^4^Departments of Neurology, Medicine, and Behavioral Neuroscience, and Oregon Institute of Occupational Health Sciences, Oregon Health & Science University, Portland, OR, United States; ^5^TBI/Concussion Program, Orthopedics & Rehabilitation and Family Medicine, Oregon Health & Science University, Portland, OR, United States

**Keywords:** concussion, traumatic brain injury, systems science, complex, multi-scale, networks, models of injury

## Abstract

Traumatic brain injury (TBI) has been called “the most complicated disease of the most complex organ of the body” and is an increasingly high-profile public health issue. Many patients report long-term impairments following even “mild” injuries, but reliable criteria for diagnosis and prognosis are lacking. Every clinical trial for TBI treatment to date has failed to demonstrate reliable and safe improvement in outcomes, and the existing body of literature is insufficient to support the creation of a new classification system. Concussion, or mild TBI, is a highly heterogeneous phenomenon, and numerous factors interact dynamically to influence an individual’s recovery trajectory. Many of the obstacles faced in research and clinical practice related to TBI and concussion, including observed heterogeneity, arguably stem from the complexity of the condition itself. To improve understanding of this complexity, we review the current state of research through the lens provided by the interdisciplinary field of systems science, which has been increasingly applied to biomedical issues. The review was conducted iteratively, through multiple phases of literature review, expert interviews, and systems diagramming and represents the first phase in an effort to develop systems models of concussion. The primary focus of this work was to examine concepts and ways of thinking about concussion that currently impede research design and block advancements in care of TBI. Results are presented in the form of a multi-scale conceptual framework intended to synthesize knowledge across disciplines, improve research design, and provide a broader, multi-scale model for understanding concussion pathophysiology, classification, and treatment.

## Introduction

Traumatic brain injury (TBI) is a significant public health concern. The United States alone sees an estimated 2.5–3.8 million cases per year (1–4), approximately 70–90% of which are mild TBI (mTBI), also called concussion (5, 6).^1^ A recent National Public Radio poll found that one in four Americans report having suffered a concussion at some point in their lives (8). Because many of those affected do not seek medical treatment, concussion is vastly underreported (4); one study estimated that at least 88% of cases might go unrecognized (9).

Despite increased awareness of TBI—and particularly concussion—in recent years, we still lack effective means of diagnosis, prognosis, and treatment (10–12). Over 30 clinical trials of pharmaceutical products to treat TBI have failed, and the U.S. Food and Drug Administration has yet to approve a single diagnostic test or therapy for the condition (13). The total cost of these failed clinical trials is estimated at 1.1 billion dollars (14). Research is hampered by imprecise classification, methodological inconsistencies, measurement issues, and uncertainty about underlying pathophysiology.

It is estimated that somewhere between 5 and 43% of concussion patients experience prolonged somatic, emotional, or cognitive impairments lasting longer than 3 months, a state referred to as post-concussive syndrome (PCS) (15–20). A recent longitudinal study found that only 27% of PCS sufferers meeting strict inclusion criteria at 3 months post-injury eventually made a full recovery (21). Accordingly, persistent post-concussive symptoms comprise a significant health burden in modern society (15). Moreover, the reasons why some patients recover quickly and others do not remain poorly understood (21–24). Factors such as genetics, health status, biomechanics, and myriad premorbid and environmental factors all likely come into play (20). In addition, the mode of injury, clinical features, and patient experience are all highly heterogenous (18, 21, 23, 25–27). These differing recovery trajectories form the primary motivation for the current project.

Traumatic brain injury has been called “the most complicated disease of the most complex organ of the body” (28). The field of systems science offers methods for understanding such complexity, as seen in the growing field of systems medicine (29–31). In this review, we survey the concussion literature through a systems lens, highlighting the complex nature of injury and recovery, as well as limitations in the existing literature. As a first step toward building a comprehensive systems understanding of concussion, we present the current state of knowledge about concussion pathophysiology using a series of multi-scale systems diagrams and discuss how this initial effort might inform clinical practice, future research, and further development of systems models.

This project has been led by a team of systems scientists in collaboration with TBI experts from the fields of neurology, neurosurgery, psychiatry, sports medicine, rehabilitation, neuropsychology, neuroscience, and others. This non-traditional review was done iteratively through multiple phases of literature review, expert interviews,^2^ and systems diagramming. It is presented here as an example of how systems methods can enrich understanding of a complex medical issue and as the first phase of an effort to develop systems models of concussion (a future publication will present a more formalized model based on this research). It should be noted that this review does not include a systematic assessment of study quality [see Ref. (32) for such a review]. The small number of high-quality studies in this field is insufficient for understanding the big picture of concussion recovery. Systems science methods have the flexibility to incorporate various types of information, including expert opinion. This big-tent approach allows for the construction of a hypothesis model that reflects a simplified vision of how a system is thought to work. The value of such a working model is that it enables a holistic perspective and discussion at the level of whole systems and subsystems—a potentially transformative perspective for complex issues such as concussion.

## State of the Research

A 2004 World Health Organization review notes the “variable quality” of mTBI studies (33), while Cassidy (34) argues that before 2002, study quality was “poor.” In an evidence-based systematic review of the literature on prevalent indicators of concussion, Carney et al. (32) found that only 26 of 5,437 studies met their criteria for analysis. Common shortcomings found in the review include lack of a comparison group, measurement and reporting inconsistencies, and potential for bias or confound. Bigler et al. (35) argue that some neuropsychological metrics used in TBI research are not suitable for detecting mild impairment, and that type II statistical error is common. Moreover, several recent systematic reviews focusing on mTBI specifically have noted a lack of a shared methodological framework within the research community (18, 36, 37).

While many of the shortcomings identified in these reviews could be addressed by more robust research methodology, progress is also hindered by more fundamental uncertainties regarding pathophysiology and measurement of TBI. These ontological and epistemological uncertainties are compounded by heterogeneity and non-linear interactions between variables—concerns common to complex problems.

### Unknown Pathophysiology

The pathophysiology of concussion is largely unknown, although several hypotheses have emerged. Giza and Hovda (38, 39) have identified a neurometabolic cascade in the acute stage following injurious impact in animal models, and some of these neurometabolic alterations have been confirmed in humans with H1-MR spectroscopy techniques (40, 41). Diffuse axonal injury (DAI) has also been identified in animal studies and proposed as an explanatory model (36, 37, 42). Other researchers, however, suggest that DAI is more likely involved in moderate to severe TBI cases, while traumatic axonal injury, which is more capable of repair, is likely to be prevalent in concussion (43). Complicating matters further is a significant literature demonstrating that the biomechanics and neurophysiology of impact injuries differ significantly from those underlying blast or penetrating concussive injuries, leading many researchers and clinicians to treat them as separate conditions (44–46). A growing body of literature examines alterations in functional connectivity networks in concussion, and how network changes over time might influence recovery trajectories and outcomes (47–52).

Cellular metabolic dysfunction immediately following injury initiates a vulnerability window; if a second concussion occurs within this window, significant further damage can occur, a phenomenon described as *second impact syndrome* (38, 53, 54). Questions about prognosis and post-injury vulnerability are of particular concern in military and athletic contexts, where social pressure exists to return to combat or play (55, 56).

No single definition of concussion is accepted across disciplines, although several are available (7, 32, 57–59). The consensus NIH definition of TBI as “an alteration in brain function, or other evidence of brain pathology, caused by an external force” (60) lacks specificity for concussion. Other definitions tend to focus on mode of injury or clinical parameters, and in sports are closely linked with guidelines for return to play (61, 62). Disagreement and uncertainty also exist about the threshold for diagnosis, or how to examine the outcome of multiple subconcussive blows to the head (63–65). However, research does support that chronic cellular dysfunction and repeated head injury can cause chronic traumatic encephalopathy, a disease often present in athletes as well as combat military personnel (66, 67).

### Heterogeneity

Many of the research shortcomings mentioned earlier can be traced to inadequate accounting for patient and injury heterogeneity and variability in clinical identification and diagnosis, which is found in several aspects of concussion (13, 23, 27). First, the mode of injury is highly heterogeneous. Traumatic biomechanical forces in the brain can occur from direct (to the head) or indirect (to the body) impact (e.g., sports, workplace accidents, and violent trauma), fast acceleration or deceleration forces (e.g., whiplash and motor vehicle accidents), or intense changes in pressure (e.g., blast exposure) (68), each inducing distinct parameters of parenchymal displacement (45, 46, 65). Biomechanics and other injury characteristics further interact with individual variation in physiology (particularly idiosyncrasies in brain topography and connectivity), along with other personal characteristics such as age (69), sex (70–72), pre-injury diseases and medications (73), and genetics (74, 75).

Concussion patients suffer myriad complaints—headache, disorientation, language impairments, loss of consciousness, mood disruptions, cognitive deficits, sleep disorders, sensitivity to light and sound, and problems with balance or gait, among others—although not all symptoms may be present in every case (18, 32, 76). Indeed, no impairment is common across all cases or all modes of injury. Loss of consciousness, once widely thought of as characteristic and diagnostic of concussion, is now understood to be present in only 1–14% of cases [(32); see Ref. (77) for discussion]. The severity and duration of each of these impairments varies and is largely unpredictable. Function in school, work, and social relationships can also be compromised, ranging from stress to disability. In addition to heterogeneity in signs, symptoms, and deficits, the factors influencing recovery—such as adherence to treatment, amount of social support, behavioral adaptation, cognitive reserve, and psychological resilience—also vary widely between individuals (27, 78, 79). Due to the heterogeneity and complexity seen within concussion, it is likely that a wide variety of destructive and restorative processes are at work following injury, some at the cellular level and others at larger scales.

At all levels of severity, TBI is increasingly recognized to be a chronic condition or ongoing disability, rather than an isolated event or fixed injury, further complicating definition and classification (7, 18, 80). Identification of any specific fixed pathophysiology therefore needs to account for dynamic evolving changes occurring over days, weeks, months, or even years. Indeed, the question of how to identify proper time points for diagnosis, measurement, or recovery trajectory has been a recurring theme among reviewers of failed clinical trials (12, 13, 58).

The types and extent of injuries currently included under the umbrella of concussion are so different from one another that a diagnosis of “concussion” alone is not very useful for informing treatment (27). Patient and injury heterogeneity, along with the lack of a common known pathophysiological explanation, raises the question of whether multiple distinct etiologies are in fact present. Sequential neuroimaging or other metrics may eventually enable characterization of subgroups defined by disease etiology, although this remains to be seen (81–83).

Even when promising avenues of research have been identified, translation to classification and treatment have fallen short, and disagreement continues over appropriate inclusion and exclusion criteria for concussion (13, 35, 84). For example, questions remain as to whether the mechanism of injury (e.g., blast or impact) or context of injury (e.g., football game, car accident, or fall) should be stratified in trials (22). Overly narrow inclusion criteria—such as only including individuals who have had a loss of consciousness, for example—artificially reduce heterogeneity (32), while overly broad criteria risk the inclusion of non-concussed or more severely injured persons. Highly variable subject groupings and lack of control groups also make studies incomparable, which significantly hinders meta-analysis and systematic review. Synthesis projects in turn are critical for creating an evidence-based definition of concussion and classification of TBI, creating a mutually dependent scenario that hinders further progress.

### Measurement

Uncertainty about the nature of concussion is compounded by problems with measurement and classification. When a TBI is suspected, classification is determined by the Glasgow Coma Scale (GCS) (85), which categorizes all TBI—from concussion to coma—on a single spectrum of mild/moderate/severe (86). This ordinal scale is based solely on measures of arousal, while assessments used to measure recovery in the clinic are diverse and often continuous. Although the acute clinical care of moderate to severe TBI patients has benefited from use of this scale, the same is not true for concussion (87), for which arousal is less informative and the GCS fails to predict recoveries or outcomes. Hack and others have argued that categorizing TBI using the GCS is “the equivalent of describing cancer as mild, moderate, and severe and then expecting that one treatment will cure all cancer” (13).

Currently, clinically practical *in vivo* neuroimaging tests sensitive to the type of structural tissue damage present in concussion remain lacking. When TBI is suspected in acute clinical settings, computed tomography and conventional magnetic resonance imaging (MRI) may be performed to identify trauma-related abnormalities such as skull fracture, brain edema, or intracranial hemorrhage. However, in concussion, such abnormalities are uncommon, and positive neuroimaging findings fail to predict long-term outcomes (43, 48, 88).

While diffusion tensor imaging (DTI) has been widely used as a measure of *in vivo* white matter integrity (66, 89), DTI metrics are also influenced by and reflect various neuropathological changes including neuroinflammation and dynamic variation, depending on time post-injury and potential reparative influences during recovery (90, 91). Two recent systematic reviews of DTI studies of concussion in humans, with inferences about *in vivo* axonal injury and integrity in human subjects, emphasized a lack of high-quality data, missing control groups, and discrepant analytic techniques in the existing literature (36, 37). Few studies met all inclusion criteria for either analysis. Asken et al. (36) concluded that while DTI is sensitive to a wide range of group differences, it currently lacks the specificity necessary for meaningful clinical application in concussion. To address these limitations, more recent studies have included larger and more diverse sample sizes and controls not meeting criteria for mTBI but with either neuropsychiatric diagnoses or other health/injury-related problems (92–94). Accordingly, future DTI studies hold considerable promise in overcoming the limitations mentioned earlier and providing objective neuroimaging correlates of brain injury and outcome.

Other methods have been used in attempt to measure and classify concussion. Event-related potentials (ERP) and quantitative EEG (QEEG) provide a direct window into the neurophysiology of the injured brain. Advantages compared to neuroimaging include portability, and high temporal and reasonable spatial resolution. QEEG has long been proposed as a potential means to aid in the diagnosis and prognosis of TBI of all severity grades (95–98). ERP, in particular somatosensory-evoked potentials, have also been investigated in both animal models and human subjects with TBI, but its clinical utility is still controversial (99, 100).

Considerable effort has been devoted to identifying serum biomarkers to identify methods for diagnosis, vulnerability, recovery, and outcome, but the clinical utility of these measures also remains limited at present (101). In particular, promising research into glial fibrillary acidic protein and ubiquitin C-terminal hydrolase-L1 is being conducted, but so far neither marker have been shown to reliably predict concussion recovery (102, 103).

### Toward New Approaches

Rosenbaum and Lipton (27) suggest that “ultimately, outcome will probably be most reliably predicted based on a complex system of clinical, pathological, and imaging variables.” Some have thus turned toward “big data” approaches. New attempts are underway to assemble large TBI data sets, both through multisite studies (104) and the compilation of existing data into shared repositories (105). For example, a recent collaboration between the National Collegiate Athletic Association and the US Department of Defense uses the Federal Interagency Traumatic Brain Injury Research (FITBIR) database to compare clinical and neurobiological recovery after concussion in student athletes and military personnel (106). These efforts are still in the early stages and face obstacles—including inherited and even amplified problems with constituent data sets—and are further complicated by the diverse perspectives of the many stakeholders involved, which include athletic and military organizations, universities, and others. Progress has also been made in establishing common data elements (107) to increase compatibility of data sets.

Group collaboration is complicated by the fact that concussion crosses multiple domains and contexts and does not fall within one medical specialty. Disparate specialties offer alternative perspectives and hypotheses about pathophysiological mechanisms, what constitutes recovery, and how to measure progress.

These concerns indicate a lack of a shared explanatory model, or idea of how concussion “works.” At issue is how we understand the full spectrum of brain injury (if it is indeed a spectrum), and how uncertainty and heterogeneity interact at various points. Many of the obstacles faced in research and clinical practice related to concussion, and TBI more broadly, ultimately stem from the complexity and heterogeneity of the condition itself. A systems perspective therefore could facilitate the creation of a shared explanatory framework—known in the systems literature as a *mental model* (108).

## Systems Approaches to Medicine

In the past decade, systems approaches to medicine have emerged to address the complexity seen in conditions such as diabetes, cancer, and heart disease (29–31, 109). Although several approaches exist, they all distinguish themselves from reductionist methods. While reductionist science is critical for answering well-defined empirical questions, it is less equipped to address questions involving greater uncertainty, complexity, and heterogeneity. In contrast, systems approaches to medicine seek to incorporate—rather than control for—the dimensions of context, space, and time (29). They emphasize the inclusion of all relevant factors to understand the function of the whole and recognize that system behavior is strongly influenced by causal structure (110, 111).

The application of systems biology to medicine has resulted in a growing area of research based on the “biology as information science” paradigm (31). This research—referred to as P4 medicine, personalized medicine, precision medicine, systems medicine, or systems biology—uses large amounts of high-throughput data (often genomic, proteomic, and other “omic” data) about an individual to develop personalized diagnosis and treatment. This approach has been used successfully for cancer and other diseases (112) and works best for diseases with strong intrinsic causal factors or known pathophysiological mechanisms (113). Other data-driven approaches, such as machine learning (114) and reconstructability analysis (115), are currently being used to identify complex, non-linear, and multivariate correlations in concussion. However, all of these approaches depend on the availability of large high-quality data sets, which are currently lacking.

Methods from the field of systems science are also increasingly applied to medicine. Systems science methods “enable investigators to examine the dynamic interrelationships of system components which may span multiple levels of analysis (e.g., from cells to society), while simultaneously studying the behavior of the system as a whole over time” (116). Rather than creating models directly from clinical data, this approach identifies model structure and parameters based on primary sources (e.g., published literature and expert interviews). Models are constructed qualitatively and then validated against clinical data. Models produced in this way have the advantage of offering a coherent hypothesis for how a system functions. As such, this approach “opens the black box” of a system and introduces a common discussion platform for diverse disciplines or stakeholders. In a medical context, these models can offer hypotheses for the behavior of populations (useful in public health), or for pathophysiological processes. Consequently, systems models can provide an approach to address the lack of a shared mental model for concussion. These models are also well suited for identifying points of leverage or intervention in complex systems. Systems science has been used in various contexts in public health (117), such as cardiovascular disease (118), obesity (119), and drug diversion and abuse (120). Recently, system dynamics modeling was used to better understand the drivers and dynamics of depression (121).

A systems focus has also begun to emerge from within disciplines. Systems neuroscience, for example, examines how neural circuits form whole systems and subsystems within the brain that relate to function and behavior (122). A recent series of articles in this journal calls attention to ways in which TBI can be studied from this perspective (123). A review by Bigler (124) in this series discusses a systems approach for examining the TBI spectrum, with a particular focus on how neuroimaging may inform multiple levels of inquiry. Here, we apply systems science methods to better understand injury pathophysiology and recovery in concussion.

## Taking a Systems View of Concussion

We assert that many of the difficulties in concussion prognosis ultimately stem from the complex nature of the condition. While definitions of *complex system* vary, the term is widely understood to refer to systems in which the behavior of the whole is not entirely explained by the behavior of parts or subsystems (116). Complex systems often have many interrelated components at multiple scales and demonstrate non-linearity, feedback, dynamic change over time, and emergent properties (125). Rather than reducing or controlling for aspects of complexity in an issue like concussion, systems methods seek to incorporate them as properties of the system.

To take a systems approach, one must identify all relevant factors or variables in a system, articulate the relationships between those variables, and acknowledge system boundaries. Variables, relationships, and boundaries together constitute system structure, which in turn determines system behavior. This determination is not always straightforward; indeed, in complex systems, certain causal structures result in counterintuitive non-linear feedback and emergent behavior. Examining how behavior changes dynamically over time in turn gives insight into system structure and provides utility to stakeholders—particularly researchers and clinicians in the case of concussion. Choices regarding indicator variables and system boundaries are determined by the problem or question that drives the modeling effort and are therefore to some degree value-laden and influenced by the perspective of the modeler. As such, it is good practice to include experts and stakeholders in the modeling process.

Building a model of a complex system often requires a considerable amount of information. The modeler needs to know which variables to include and how to measure them, how to quantify the relationships between those variables, and how best to describe system behavior over time. Also required is data against which to verify the model. For systems with known variables, undisputed structure, and good reference data, models can be built with solid empirical support. Missing data or uncertainty or disagreement about system structure, however, limits one’s ability to build such a model. In these cases, understanding the complexity of the system also entails acknowledging and incorporating this uncertainty through qualitative approaches. Being able to incorporate expert judgment into a model of hypothesized causal structure allows for the examination of system-scale behavior even if variable-level data is sparse. It also allows us to examine the extent of agreement and identify gaps in existing knowledge and inconsistencies in mental models.

Here, we provide a conceptual framework that identifies system boundaries and variables influential to concussion recovery. This is a preliminary step in the modeling process and is intended to guide further inquiry into the complex nature of concussion recovery, including the construction of formal models. The lack of a shared mental model for the pathophysiology of concussion, disagreement over definition, diagnosis and recovery, failed clinical trials, and unavoidable patient and injury heterogeneity all support the need for a common conceptual framework. Such a conceptual framework should be flexible enough to account for individual differences while providing enough structure to enhance understanding, and may serve as a useful decision support tool in clinical and research settings.

## Concussion at Multiple Scales

**Figure 1:**
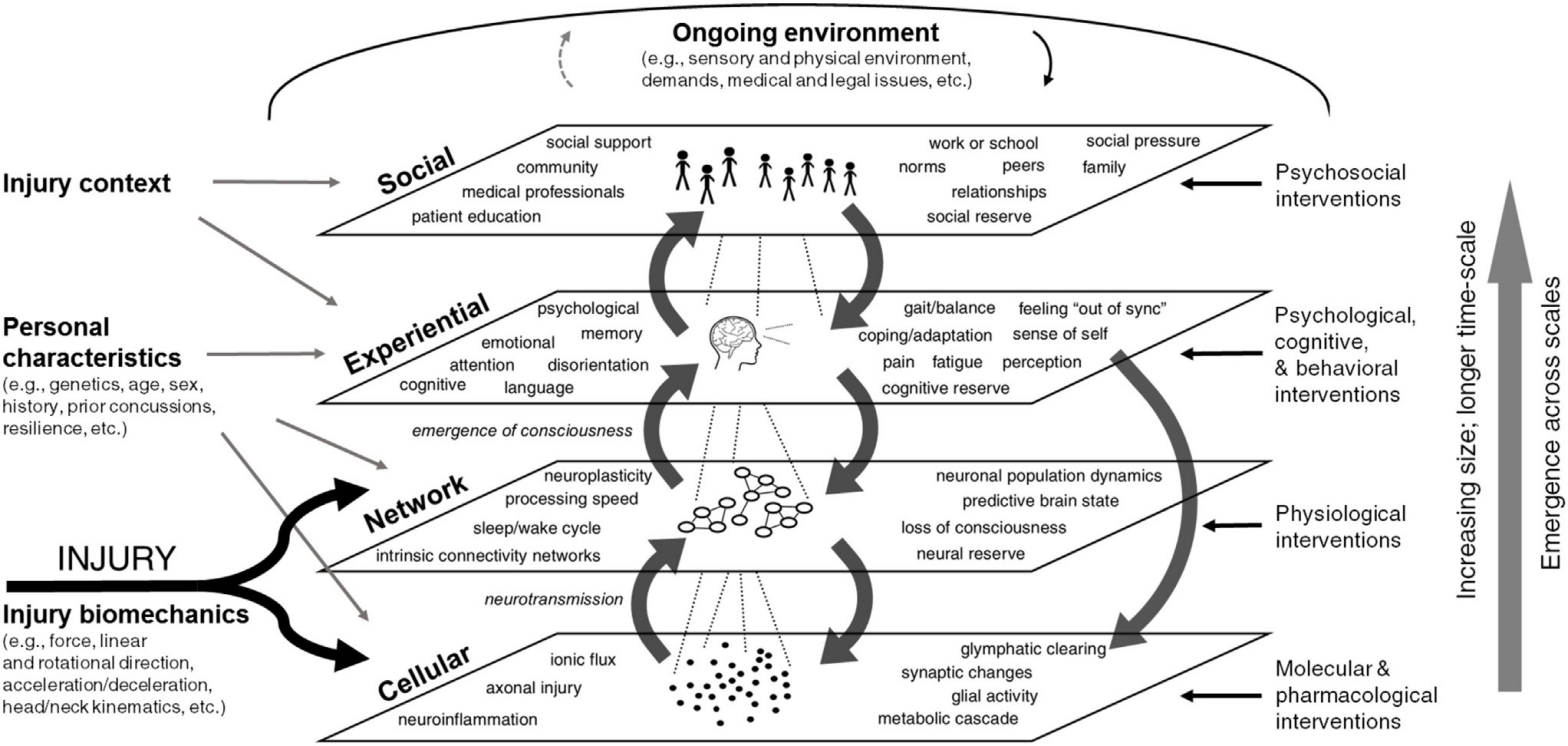
Multi-scale framework for concussion. Factors influencing concussion pathophysiology and recovery are shown across four nested emergent scales: cellular, network, experiential, and social. Endogenous factors—those that affect and are affected by other factors in the system—are included inside the scale boxes. Exogenous system drivers that act upon the system are shown at the perimeter. On the left are exogenous factors present at the time of injury (e.g., injury phenomena and biomechanics, personal characteristics, and injury context), while interventions on the right and top margins impact the system dynamically during the recovery process. Aspects of the ongoing environment influence factors at all scales. Feedback exists within and also between scales. Medium gray arrows indicate cross-scale interactions. Factors show emergence, increasing size, and longer time-scale moving up from the cellular to social levels. A team of systems scientists produced this diagram based on literature review, interviews with researchers and clinicians, and iterative review by subject matter experts.

Systems approaches conceptualize complex biological systems as consisting of elements at multiple nested scales (126, 127). Figure 1 shows key aspects of concussion injury and recovery mapped across four scales: cellular, network, experiential, and social. Factors endogenous to the system—those that affect and are affected by other factors in the system—are included inside the scale boxes. Exogenous factors that drive the system from the outside are indicated at the margins, including those present at the time of injury (injury phenomena and biomechanics, personal characteristics, and injury context), as well as interventions that take place during recovery. Aspects of the ongoing environment influence factors at every scale and can themselves be affected. It should be noted that Figure 1 is a static representation of a dynamic, interconnected system and as such does not capture the full complexity of the system over time. It also does not capture the full extent of variables relevant to concussion recovery, nor the individual heterogeneity seen for these variables.

### Cellular Scale

This scale includes cellular processes (along with molecular subprocesses) most critical to concussion. Ionic flux leads to metabolic dysfunction and energy imbalances, which in turn contribute to additional neuroinflammatory responses to injury (38, 39). This cascade impairs neurotransmission in individual neurons. An additional cause of impaired neurotransmission is axonal injury caused by mechanical shearing and stretching forces (128, 129). Also impacted at the cellular scale are vascular integrity and glial cell function, which exacerbate existing damage and further increase neuroinflammation (68). A recently described gliovascular pathway for clearance of toxic proteins and metabolic wastes, the glymphatic system, may also be impaired in TBI and be exacerbated by sleep disturbances (130, 131).

Multiple feedback loops exist within the cellular level. For example, axonal injury increases further neuroinflammation *via* microglial activation (43, 132, 133). These variables and their interrelationships may be caught in cycles of regeneration and repair, or degeneration and dysfunction (128). Note that effects at this scale are not limited to the acute phase of injury; rather, biochemical and cellular changes can occur days and even years after a traumatic event, even after clinical symptoms resolve (134).

Figure 1 shows that injury characteristics—particularly injury biomechanics—directly impact the cellular level. Injury biomechanics include the objective measures of force, direction, and physics of impact as they are transferred through the skull into the brain. Personal characteristics such as genetics, age, and sex can also influence cellular-scale processes. For example, sex differences in neck stability render female athletes significantly more likely to experience a concussion compared to male athletes in the same sport (71, 72, 135). Pharmacological interventions (e.g., medications to address headache or comorbid muscle pain and tension) can also modulate the cellular state on an ongoing basis during the recovery process.

Overall, well-functioning processes at the cellular scale allow for communication between individual neurons. The net effect of the cellular level constitutes effective neurotransmission—the unified orchestration of cells in a particular cellular microenvironment, including not only individual neurons but also their associated microvasculature, glial cells, and various cytostructural matrix proteins in the intracellular and extracellular space. At the cellular level, successful neurotransmission is what allows for a neuron to communicate with other neurons and function in modular neuronal assemblies (136–138).

### Network Scale

Neurotransmission leads to the emergence of neuronal networks from the synchronized activity of many neurons either simultaneously or in patterned sequences. These neurons may send information *via* direct electrical or molecular signaling (structural connectivity networks) or through temporally synchronized activations or deactivations (functional connectivity networks). Since there is often no relation between the location or extent of visible focal injuries and symptoms, performance, or outcome in concussion (139), and individual cellular processes cannot yet be easily examined non-invasively in humans *in vivo*, an examination of networks may provide the missing link between overt behavior and individual molecular or cellular trauma (47, 48).

By merging structural connectivity analyses (e.g., DTI) with functional measures (e.g., fMRI), researchers have been able to reliably identify intrinsic connectivity networks—specific groups of brain areas that temporally synchronize to support given functions that may be close or far apart and may be activated or deactivated as individual nuclei or entire regions (137, 138, 140–142).

An emerging literature demonstrates structural and functional reorganization in network connectivity within and between networks in concussion, particularly in the acute to early timeframe (36, 37, 48, 140, 143–150). Evidence suggests that network alterations are particularly prominent in frontoparietal regions following concussion (48–50, 151–153), which may directly slow information processing (154). Electrophysiological findings demonstrate slowed information processing and changes in neuronal encoding and function over time as a result of brain injury (155). Notably, several studies have now identified alterations in resting state networks, a global interhemispheric and intrahemispheric network of brain regions that are consistently turned off during task-related activities while remaining more active during rest (142, 156). In concussed patients, these resting state networks are disrupted and unable to completely turn off, thereby affecting self-awareness, working memory, attention and performance (26, 49, 146, 147, 157–159). For a more thorough review of neuroimaging and concussion see Bigler et al. (48) and Hayes et al. (144).

**Figure 2:**
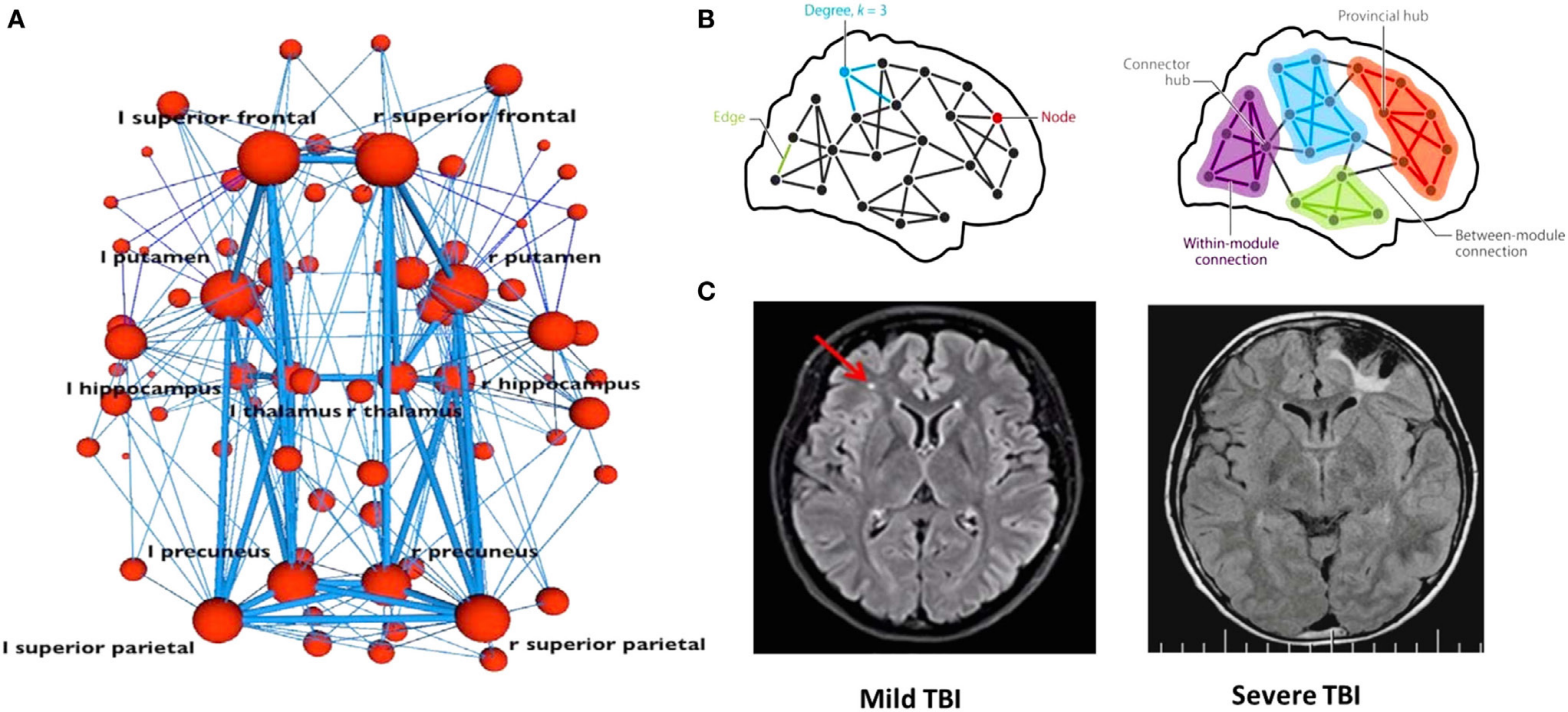
Concussion at the network scale. When investigating how information passes through a network **(A,B)**, a node may represent neuronal activations, or network properties of gray or white matter in a given location. Connections between nodes may represent structural white matter tracts (i.e., groups of axons), temporal synchronization, or other functional relationships. Major hubs [shown in panel **(B)**] refer to regions that play a central role in connectivity and information processing. For any given structural or functional connectivity network, major hubs may be contrasted with less important hubs and peripheral nodes and edges. Comparison of magnetic resonance imaging scans from mild and severe TBI patients is shown in panel **(C)**. The smaller the site of injury, the less likely the damage is to significantly interrupt information processing within a given network. In the concussed patient, if the injury impacts a peripheral small node, there might only be a minimal disruption in function, especially if the functional connectivity needed for recovery is restored via rerouting. In the severe TBI case, such workarounds will not be possible as the damage is too extensive. A network approach illustrates the possibility that severity or prognosis may be based on extent of network damage. Panels **(A,B)** were borrowed from Ref. (138), and panel **(C)** was borrowed from Ref. (124). Used with permission.

The neurobehavioral and neurocognitive sequelae of concussion relate to how and at what levels networks become affected (see Figure 2). In the concussed patient, damage to a major neuronal hub—which plays a central role in connectivity and information processing—can have a significant impact. But if the injury impacts a peripheral small node there may potentially be only a minimal disruption in function, especially if functional connectivity is restored by rerouting information through another intact pathway. The brain’s ability to reroute will depend on neuroplasticity, neural reserve, and other network properties of structural and functional connectivity [see Ref. (136, 138) for a description of cortical communication dynamics].

Neural reserve reflects the amount of brain damage that can be sustained before reaching a threshold for clinical expression; it is a passive measure correlating with early childhood education and socioeconomic status and may be represented by brain size or overall synapse count (78). If a person has high neural reserve, information signals may bypass areas of tissue damage easily to find another route to their intended destination, thereby reducing or even preventing any noticeable functional impairment. The smaller the site of injury, the less likely it is that the damage will significantly interrupt information processing within a given network. MRI scans from mild and severe TBI patients (Figure 2C) illustrate the possibility when taking this approach that severity or prognosis may be based on extent of network damage.

Significant alterations in timing or neuronal synchronization at the neuronal population level may impede activation or deactivation of entire brain regions, or disrupt global processing. Even the slightest change in timing can significantly disrupt network coordination of cognition (160), attention (152), and even the sleep/wake cycle (161, 162). It is important to note that the effects of brain injury are not solely determined by the area of damage; rather, subtle changes in timing or processing speed can have large effects downstream (160). For example, networks underlying postural control and gait require constant updating and integration of current sensorimotor and vestibular information for normal balance and walking to occur (163). In the concussed patient, these networks might not be damaged *per se* but instead are simply functioning at slower processing speeds, leading to inaccurate updating and therefore functional impairments in gait. Indeed, mapping and understanding the complexity of temporal synchronization in the brain is critical to defining damage and predicting recovery.

Further study of temporal network dynamics will be critical for understanding the heterogenous symptoms and impairments that arise in response to concussion [see Ref. (160) for a review]. Since networks provide the link between cellular insult and the felt experience of concussion, focus on specific types of network damage may help clinicians guide treatment. For example, Ghajar and Ivry (152, 164) have argued that TBI selectively impairs attentional networks in the cerebellum, frontal lobe, and parietal lobes involved in the generation, maintenance, and precise timing of predictive eye movements. These distributed networks are hypothesized to allow the brain to be predictive (as opposed to reactive) with regards to external visual stimuli. In the injured brain, disrupted timing in this network may shift the brain into a reactive mode, causing impairments in visual smooth pursuit (or predictive eye tracking), which one day may be used as a diagnostic marker for concussion. Indeed, examination of networks underlying automatic eye movements is a promising area of TBI research (165–167).

However, it should be noted that despite widespread optimism that network science will identify neuroimaging biomarkers for concussion (47, 48, 50), this research still faces many of the obstacles of uncertainty and heterogeneity outlined earlier, as well as its own methodological challenges. Networks are dynamic and constantly changing, which complicates determinations of baseline. Moreover, researchers are only beginning to understand the relative contributions of particular networks to individual conscious experiences. Even seemingly singular experiential events (e.g., pain) involve the coordination of multiple networks (168). Delineating how to categorize and compare networks (e.g., unimodal versus crossmodal and inter- versus intrahemispheric), as well as connectivity between networks (i.e., hyperconnectivity versus hypoconnectivity) will be critical to understanding network changes associated with concussion.

### Experiential Scale

**Figure 3:**
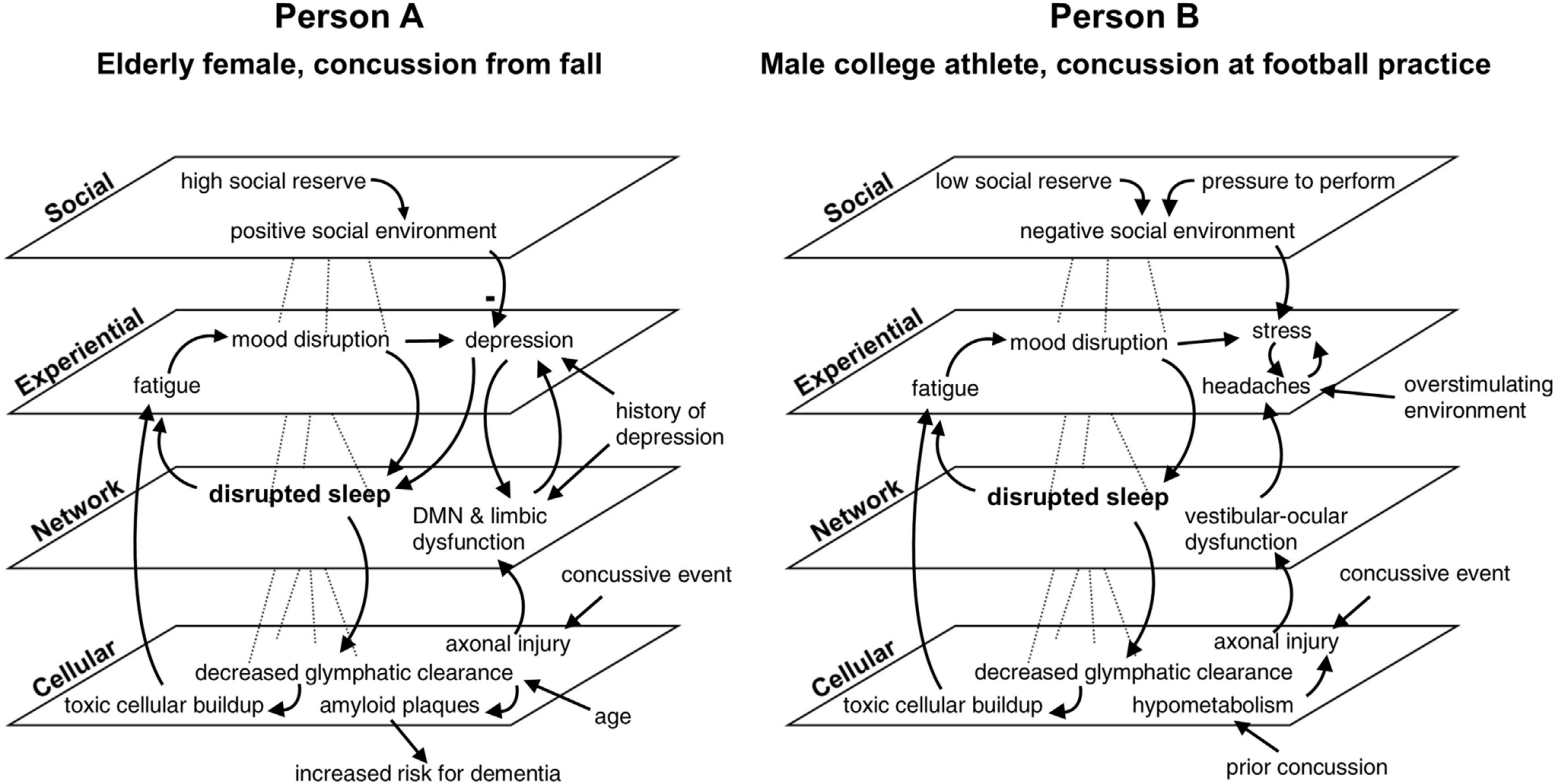
Multi-scale map of factors influencing recovery from concussion in two cases. In this comparative hypothetical example, two individuals present with sleep disruption of the same magnitude at the same time point after injury. Additional variables differentiate the cases. Factors are mapped across four nested scales and are linked with arrows indicating causal influence. The minus sign in the diagram for person A signifies a reduction in depression. While both persons show the same symptom (i.e., sleep disturbance), outcomes differ between the two cases according to distinct profiles of feedback relationships with other variables. Disrupted sleep will result in clinical depression for person A if left untreated, placing her at increased risk of dementia, given her medical history and age. Person B might develop persistent post-concussive symptoms if the compounding effects of stress and headache are not addressed. Default mode network is abbreviated DMN. This example illustrates the utility of considering scale and feedback in the clinical care of concussion.

Networks working in concert with one another enable the emergence of consciousness, which introduces variables at the experiential scale. This is the level at which an individual experiences the concussion as it plays out in time and awareness, through symptoms such as headache or disorientation. Dysfunction of networks in concussion influences psychological, emotional, and cognitive states, causing such problems as memory and language impairments, mood disruptions, and gait/balance issues (37, 47, 124). For example, changes in networks subserving the sleep/wake cycle manifest in felt experience as noticeable disturbances in attention, cognition or mood, as reflected in the hypothetical case examples shown later in Figure 3. Temporal asynchronies in neuronal communication lead to disruptions within and between brain networks that manifest experientially as cognitive dysfunction (136, 160). For example, changes in neuronal timing brought on by concussion have been shown to disrupt networks underlying smooth pursuit eye movements, disturbing perception and leading to self-reported feelings of being “out of sync” (152).

Impairment in self-awareness is common after TBI and may occur due to decreased functional connectivity within frontoparietal control networks that are also associated with deficits in attention and performance monitoring (49, 164). This disruption in self-awareness can impact a person’s ability to successfully report on her own symptoms and can influence how she perceives and interacts with her environment. These disturbances are influenced by the individual’s premorbid emotional and psychological functioning (169, 170). In patients with comorbid injuries or a history of chronic pain, comorbid pain or muscle tension can disturb perception further, increasing psychological distress and interfering with daily function. Several experts interviewed in the course of our research emphasized how perception of one’s injury and expectations for recovery can profoundly shape experience. Also influential is how a person copes with or adapts to their symptoms and deficits. Coping and adaptation skills may be learned but can also be facilitated at the neuronal level by cognitive reserve. Influenced by neural reserve, cognitive reserve impacts one’s ability to adjust to brain injury through various compensatory mechanisms or adaptations [see Ref. (78) for a review].

Personal characteristics such as psychiatric history (e.g., mood disorders or PTSD) and personal resilience also influence how people experience concussion (79). These factors are also thought to influence the brain and body’s response to injury at the cellular and network levels (171). The injury context—the setting in which the injury took place—may impact one’s perception of the injury, as well as determine how quickly they are evaluated, receive treatment, and return to play or work. In addition, the demands of the environment shape recovery in an ongoing way, particularly in the presentation of symptoms and efficacy of interventions. For example, a person living in a combat zone or other high-stress situation might not be able to take as much time for cognitive rest as a person in a low-stress home environment. Tasks required to obtain medical care or pursue litigation can also be stressors. Physical or sensory aspects of the immediate physical environment greatly influence certain symptoms, such as headache and cognitive fatigue. Individuals with light sensitivity may only experience their symptoms in lighted environments, but not when they are sitting in a dark room. Thus, to understand the heterogeneity, uncertainty, and complexity in the conscious, felt experience of concussion, we must understand how these variables appear, disappear, and are modulated by personal characteristics, history, and context.

### Social Scale

A comprehensive map of concussion necessarily includes the social level of the system. This scale encompasses the manner in which relationships and interactions with other people impact an individual’s injury or recovery, and acknowledges that many aspects of concussive symptoms are determined intersubjectively. While these factors exist externally to the individual, they are included here as endogenous to the system of injury because they substantively influence—and are influenced by—properties of the individual. The robustness of social relationships with family and friends, as well as the support provided by these relationships, can play a significant role in recovery from TBI (172, 173). Support is needed to meet logistical needs (e.g., transportation and household tasks), as well as emotional needs, which may be elevated due to the psychological and emotional toll of migraine, sleep disruption, and cognitive or language impairments.

Quality of social relationships has been shown to strongly influence prognosis and recovery in cancer (174) and is likely to have similar effects in TBI. Similarly, research in cancer populations has repeatedly demonstrated that social factors, particularly physician communication styles, are powerful determinants of outcome and symptom expression (175–178). Given the complexity of concussion as an oftentimes “invisible” injury (179), it stands to reason that social norms, expressions, and communication styles surrounding the condition likely contribute to its observed heterogeneity and should not be ignored. Indeed, patient-directed educational materials about concussion, including pamphlets and information sheets received at medical discharge, may profoundly impact symptoms and deficits (181).

A person’s engagement in group settings such as work or school can also play a role (positively or negatively), as can broad social factors such as community and norms. Lack of support and pressure to perform or meet social demands can negatively influence recovery. In parallel with neural and cognitive reserve, we use the term *social reserve* to indicate the extent to which an individual’s social relationships serve to buffer the negative effects of the injury and promote coping and healing. The larger social context of the injury also shapes social dynamics. For example, a playoff game and a motor vehicle accident are contexts with different social demands. During recovery, the ongoing environment continually shapes social contexts and social expression.

The feedback from the experiential scale to the social scale is significant; a person experiencing post-concussive impairments in cognitive functioning, mood, and working memory may be compromised in his ability to function successfully in social environments, which can weaken social relationships and reduce engagement in group settings (181). Headache and the need to avoid overstimulating environments provide further obstacles to maintenance of social relationships. When a concussion is sustained in sports or military contexts, dynamics of inclusion and duty may alter when and how symptoms are expressed.

### Emergence across Scales

These scales show emergence in the sense that the interaction between elements at one scale gives rise to entities at a larger scale (182). Definitions of *emergence* vary widely. In a commonly cited definition, Goldstein refers to emergence as “the arising of novel and coherent structures, patterns and properties during the process of self-organization in complex systems” (183). Networks have distinct emergent properties of their own, but cannot exist without functioning individual neurons and circuits. Likewise, a person’s conscious experience depends on the functioning of underlying brain networks. When damage to networks results in loss of consciousness, for example, all phenomena above the network scale are temporarily suspended.

When functionality is compromised but not completely suspended, the effects might only be evident at higher levels. While slight disruptions in network timing would not be sufficient for networks to go offline, they could, for example, cause a person to feel “out of sync” or experience balance problems. In a similar vein, certain psychological impairments might not be noticed by the concussed individual but are evident to others close to him. The scales are nested in the sense that smaller-scale phenomena take place within the context of phenomena at larger scales. From bottom to top, the size of the elements at each scale increases, as does the relevant time frame at which action is observed.

### Feedback between Scales

Importantly, these scales do not exist in isolation, nor are their interactions limited to upward linear emergence. Factors influencing concussion recovery are highly interconnected and can show feedback, which is evident when the output or outcomes influenced by a variable ultimately return to and influence that variable. For example, headaches can cause increased stress, which can cause additional headaches (184). This kind of feedback results in non-linear system dynamics. Such system behavior can be counterintuitive and difficult to predict. Most statistical analysis methods cannot easily account for non-linearity, which may partly explain why TBI research has yet to discover measures that accurately predict outcome (124).

Figure 3 shows how one symptom—disrupted sleep—exists within a constellation of factors influencing the recovery of two hypothetical individuals. Both individuals suffered concussions and experienced post-concussive symptoms at 3 months post-injury. In both cases, disrupted sleep is at the center of two feedback loops: one in which sleep disruption causes fatigue and mood disturbance, which further impacts sleep, and another in which decreased glymphatic clearance results in toxic cellular buildup, exacerbating fatigue. The other factors at play in each case are different: for person A, an elderly woman who suffered a concussion from a fall in her home, a history of depression as well as additional network damage to the default mode network and limbic systems put her at risk for acute mood disruption, and lead to clinical depression. Tendency toward depression in this case is reduced, however, by strong social support and social reserve. Person A’s advanced age also reduces the efficiency of glymphatic clearing, which puts her at increased risk for dementia *via* amyloid plaque deposition. Person B, a young man injured during football practice, experiences stress and headaches, which compound one another and worsen in environments with loud noises, bright lights, or prolonged screen time. These symptoms combined with vestibular–ocular network dysfunction prevent him from fully returning to play and school. A recent prior concussion caused residual hypometabolism, which increased the amount of axonal damage he suffered during the second injury. Because he is away from home and may not have many supportive relationships outside of football, he is considered to have low social reserve which, combined with social pressure to return to play, creates a negative social environment that increases his stress. Outcomes for both patients are potentially negative without early intervention, but the unique constellation of factors in both cases produces differing profiles of symptoms and deficits.

The feedback relationships formed by these interlinkages exert influences that persist over time. Articulating these dynamics can aid in understanding an individual’s trajectory of recovery and help to identify possible leverage points and interventions in a clinical setting. By considering system drivers, an astute clinician might determine that for person A, sleep disturbance and a history of depression puts her at greater risk for acute mood disruption to turn into full-blown depression and should therefore be more aggressively treated. A clinician treating person B, whose vestibulo-ocular and headache symptoms might typically be treated with medication, could be inspired by a systems perspective to also look into improving social support for his patient, or provide education about how to deal with overstimulating environments.

### Measurement and Limitations to Knowledge

Ontological differences at each scale lead to epistemological differences. Because dysfunction can happen at any scale, a wide variety of measures are used in research and clinical practice to assess injury and impairment (see Table 1). Measures at each scale often have common challenges, which are shaped by the types of knowledge obtainable at that level and methodological constraints.

**Table 1:** Assessment of concussion across scales.

Level	What is measured?	Assessment methods	Challenges
Cellular	Structure and function of neurons, glia, vasculature, and cytoarchitecture; biomarkers of tissue damage	Proteomics (e.g., glial fibrillary acidic protein); blood serum biomarkers (e.g., hemosiderin and SB-100); animal models for brain injury (e.g., LFPI and various impact models); and postmortem histological analyses	Limited translation from animal models; lack of non-invasive *in vivo* human data; and no successful Phase 3 clinical trials
Network	Connectivity, timing, and functioning of brain networks	Neuroimaging (e.g., diffusion tensor imaging, magnetic resonance imaging, fMRI, MRS PET, MEG, event-related potentials, and quantitative EEG); eye tracking; reaction time measures; balance and gait measures; neurological assessments; and sleep assessments	Neurodiagnostic limitations (feasibility and resource requirements; prohibitive cost in clinical settings); lack of baseline or matched control scans
Experiential	Symptoms; deficits in cognitive, psychological, and emotional functioning	Neuropsychological assessments; self-reported symptom logs and health history; gait and balance tests; and psychophysics (light or sound sensitivity)	Reliability and accuracy of self-report; current neuropsychological assessments not designed for concussion; and variability in self-awareness and symptom expression
Social	Signs; strength of social relationships and social functioning	Medical evaluations; informant reports; and information about context of injury	Detection accuracy; reliability of informant reports; and differential access to health care

Uncertainty in measurement is compounded by a more fundamental uncertainty about exactly *what* is being measured in the first place. Without a shared understanding of the etiology (or etiologies) of concussion, decisions about what to measure—and when and how to measure it—vary widely. This diversity in assessment presents obstacles for secondary analysis.

## Mapping the Landscape of Concussion

**Figure 4:**
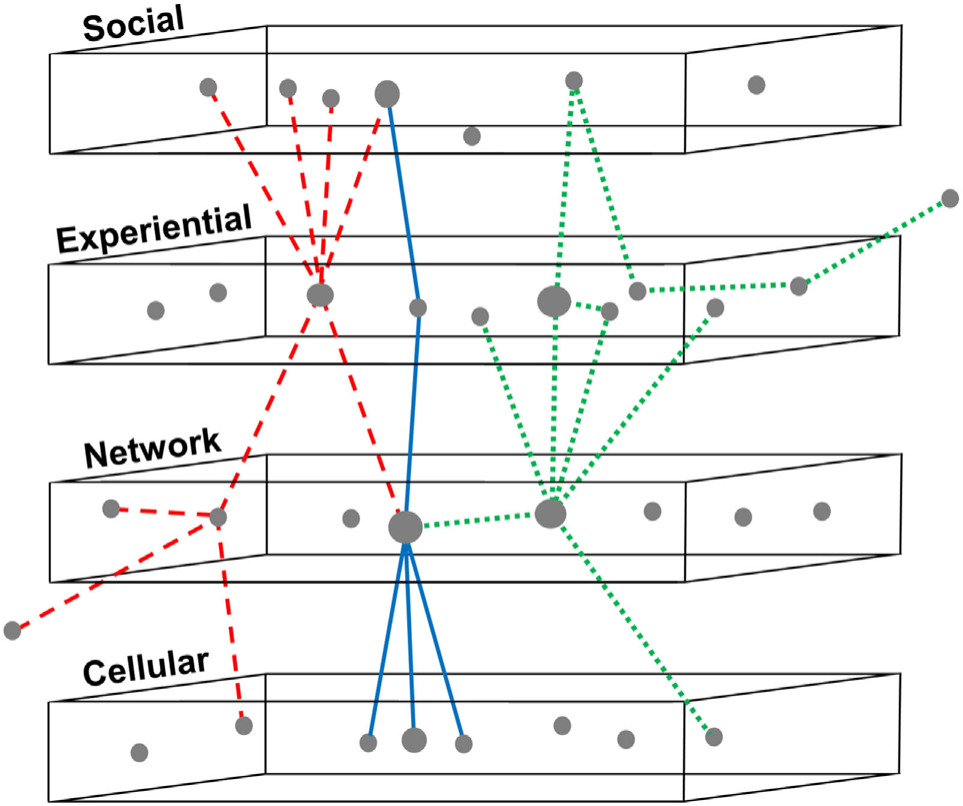
Identifying patterns of variables across multiple scales in concussion. Three subgroups are shown using dashed, solid, and dotted lines to indicate relationships between variables in concussion, mapped across four nested scales. This framework can be utilized for synthesizing existing data and knowledge in concussion, based on systematic review or big data analytics. Relationships between variables can be organized to visualize a “landscape” of factors, within which patterns or subgroups might be identified. This subgrouping would allow better phenotyping for the design, recruitment, and analysis of clinical trials and might enable reanalysis of failed drug trials to distinguish responders from non-responders. The ultimate goal of such a model is better clinical prognostication of outcomes following concussion and therefore more personalized treatments.

A multi-scale systems model of concussion can serve as a framework for synthesizing data and knowledge about correlations between variables to visualize a “landscape” of factors, within which patterns or subgroups might be identified (Figure 4). These subgroups might differ in etiology or presentation and ideally would be distinguished using one or more biomarkers. But because it is presently unknown whether heterogeneity in signs, symptoms, and deficits is intrinsic to concussion or simply an artifact of data collection or improper patient groupings, a multi-scale framework can be a first step to navigating what is observed. Organizing the “black box” of concussion into scales can allow for the identification of methodological constraints and knowledge limitations at each scale, and then aid in the refinement of research questions and implementation.

While emerging research on imaging and serum biomarkers is promising, we cannot presuppose that a single “silver bullet” biomarker will be identified. Indeed, in systems medicine, disease heterogeneity is seen as necessitating multiple markers (185). An expanded conceptual framework for concussion provides a way to contextualize possible biomarkers and see how the variables they measure are interconnected with other relevant factors.

Informed by data-driven efforts, existing clinical studies, and expert opinion, the TBI community could use this initial framework to help develop a shared hypothesis of the pathophysiology and factors influencing concussion recovery. Big data approaches can contribute to this mapping and use the framework to help interpret results. Indeed, iteration between predictive and explanatory models can be beneficial to both endeavors (186). Given the present failure of multisite clinical trials, this framework may also be used to compare data sets from multiple research sites, or contrast data from distinct modes of injury (e.g., military, athletics, and auto accidents) or time periods within recovery. Similarly, one may compare cases of better and worse prognosis to help identify variables most critical to recovery outcomes, and tailor treatments or interventions at that level.

In addition to increasing our overall understanding of this complex, traumatic syndrome, the framework may also contribute to clarifying the definition of concussion and to help develop a more nuanced classification of concussion to better facilitate personalization of treatment and to sharpen clinical trials. A systems model cannot replace a classification system, as the identification of subtypes or patterns with which to classify patient populations is necessary for clinical and research applications. But by providing a way to articulate hypothesized causal relationships in concussion, it can serve as a decision support tool for the TBI community. Because a systems model reflects the ontological structure of the phenomenon, it can serve as a foundation for future inquiry, including more sophisticated modeling efforts.

## Decision Support for Research and Practice

Systems models can serve as powerful tools for synthesizing information, which can provide decision support to researchers and clinicians by encouraging whole-systems thinking, facilitating communication across specialties, and supporting the development of shared hypotheses to identify potential treatment interventions. A student with sound sensitivity, for example, could be offered a solitary work space that would be quieter but more socially isolated, or she could be given earplugs and remain in the regular classroom. Both situations allow for more focus and fewer headaches, but the setting with more social interaction may be better for recovery. For an athlete with post-concussive concentration or attention deficits, the clinician might probe deeper about aspects of the patient’s ongoing environment to see if the deficits are due to cognitive deficits, altered sleep, distracting pain, or stress. Attempts to improve sleep hygiene, reduce pressure for return to play, manage pain, or increase cognitive rest might be helpful before or conjunction with prescribing attention-related medication.

A systems approach can also help identify unintended consequences. Suppose a patient has difficulty concentrating, headache pain, and a preexisting sleep disorder that is worsened after suffering a concussion. The physician has prescribed cognitive rest to help with concentration and exposure to stimulating environments but has not considered that reduced movement and increased downtime can exacerbate sleep problems and disrupt nocturnal rhythms. According to the above framework, the clinician has responded to symptoms at the experiential level without considering underlying processes related to sleep disruption and glymphatic clearing at the network and cellular levels. A more comprehensive approach incorporating physical exercise, cognitive rest, and behavioral sleep hygiene may be needed.

An awareness of the multi-scale nature of concussion and the key variables at each scale may help researchers identify gaps in the literature and better appreciate how individual investigations fit within the larger picture. Considering effects between levels, and drawing attention to understudied relationships, may help to sharpen inclusion and exclusion criteria for clinical trials.

Systems models—particularly diagrams—can facilitate discussion between health-care providers from different medical disciplines. Neurosurgeons and psychologists have different lexicons and research practices, for example, but both may be able communicate their knowledge using a diagram format organized according to causal or ontological structure. This kind of object—known as a *boundary object* in the science studies literature—can be used to identify research gaps and questions, synthesize a body of knowledge, and communicate with stakeholders (187). Systems models also encourage the identification of constructs that would be important to understand the system but might not be well defined in the literature. For example, in our research, the concept of *bandwidth* was repeatedly identified by clinicians as important to their patients but is not well defined in the literature. Further analysis could investigate the mechanisms behind this concept.

## Future Directions

The multi-scale framework presented in this article is the first part of a larger project applying systems science approaches to concussion. The next step will be to construct a causal-loop diagram, a more precise conceptual model in which relationships between specific system variables and feedback dynamics are made explicit (manuscript in preparation). This diagram will serve as a map of the current knowledge about concussion pathophysiology and recovery. Future work could involve developing a computational system dynamics model, which would introduce the dimension of time and allow for a closer examination of recovery trajectories.

## Conclusion

Accurate diagnosis, prognosis, and effective treatment of TBI—particularly concussion—are hindered by an imprecise classification system, a dearth of high-quality clinical trials, and lack of a shared, evidence-based working model of concussion pathophysiology and recovery within the medical community. A shared conceptual framework for concussion is needed to facilitate interdisciplinary communication and understanding of concussion, identify patterns and gaps in existing knowledge, and contribute to ongoing efforts to develop a new classification system for TBI that is more suitable for concussion diagnosis and treatment. Ultimately, our understanding of concussion will depend on the ability to account for patient and injury heterogeneity, dynamic non-linear feedback, and emergent properties intrinsic to consciousness. Systems science approaches can provide novel and useful contributions to the study of TBI and may provide a starting point for a paradigm shift in our conceptual grasp of concussion in all its complexity.

## Author Contributions

EK was the lead author for the article (text and diagrams), co-lead for conception and design, and conducted expert interviews and literature review. EP was the lead for literature review, co-lead for conception and design, contributed substantially to the article, and conducted expert interviews. EB, ML, JC, and WW provided critical review and revision of the article and contributed to conception and design. WW also conducted expert interviews and review of the literature.

## Conflict of Interest Statement

The authors declare that the research was conducted in the absence of any commercial or financial relationships that could be construed as a potential conflict of interest.

## Footnotes

^1^The extent to which *mTBI* and *concussion* can be used synonymously is debated in the literature (7). Here, we use them interchangeably. We primarily use the term *concussion* because although uncertainty exists regarding its definition, it is flexible enough to allow for a new classification system [whereas mTBI is tied to the Glasgow Coma Scale (GCS)].

^2^The authors conducted 26 semi-structured interviews, each lasting 1.5–2 h, with researchers and clinicians in the field of TBI. Nearly all of the interviews were conducted by two interviewers, recorded, transcribed, and analyzed to identify claims useful to the development of the systems diagrams. Data from these interviews provided background knowledge for the current publication and will be used in the development of future systems models.
